# Induction of Salivary IgA and IgG Responses by Parenteral PPV23 Vaccination in Older Adults

**DOI:** 10.1093/ofid/ofag273

**Published:** 2026-05-04

**Authors:** Maxime Visser, Irina Tcherniaeva, Lia de Rond, Mary-lène de Zeeuw-Brouwer, Monique W M Verschuren, Susan H S J Picavet, Marien I de Jonge, Gerco den Hartog, Anne-Marie Buisman

**Affiliations:** Centre for Immunology of Infectious Diseases and Vaccines, National Institute for Public Health and the Environment, RIVM, Bilthoven, The Netherlands; Laboratory of Medical Immunology, Radboud University Medical Center, Nijmegen, The Netherlands; Centre for Immunology of Infectious Diseases and Vaccines, National Institute for Public Health and the Environment, RIVM, Bilthoven, The Netherlands; Centre for Immunology of Infectious Diseases and Vaccines, National Institute for Public Health and the Environment, RIVM, Bilthoven, The Netherlands; Centre for Immunology of Infectious Diseases and Vaccines, National Institute for Public Health and the Environment, RIVM, Bilthoven, The Netherlands; Centre for Nutrition, Prevention and Health Services, National Institute for Public Health and the Environment, RIVM, Bilthoven, The Netherlands; Julius Center for Health Sciences and Primary Care, University Medical Center Utrecht, Utrecht, The Netherlands; Centre for Nutrition, Prevention and Health Services, National Institute for Public Health and the Environment, RIVM, Bilthoven, The Netherlands; Laboratory of Medical Immunology, Radboud University Medical Center, Nijmegen, The Netherlands; Centre for Immunology of Infectious Diseases and Vaccines, National Institute for Public Health and the Environment, RIVM, Bilthoven, The Netherlands; Laboratory of Medical Immunology, Radboud University Medical Center, Nijmegen, The Netherlands; Centre for Immunology of Infectious Diseases and Vaccines, National Institute for Public Health and the Environment, RIVM, Bilthoven, The Netherlands

**Keywords:** antibodies, mucosal immunity, older adults, PPV23, saliva

## Abstract

Mucosal immunity restricts *Streptococcus pneumoniae* colonization, thereby reducing the risk of transmission and disease. This is the first study to demonstrate that parenteral 23-valent pneumococcal polysaccharide vaccine (PPV23) vaccination induces salivary IgA and IgG responses in older adults, aged 72–79, an age group at increased risk for pneumococcal disease.

*Streptococcus pneumoniae* remains a major cause of invasive pneumococcal disease (IPD) and community-acquired pneumonia (CAP) in older adults [1]. Colonization of the upper respiratory tract is a prerequisite for disease and a principal reservoir for transmission [2, 3]. A study conducted in the Netherlands reported pneumococcal carriage in 8%–20% of asymptomatic community-dwelling adults aged ≥ 65 years, depending on the detection method used [4, 5].

In a controlled human infection study, 13-valent pneumococcal conjugate vaccine administration reduced colonization, which was associated with mucosal IgG and not IgA [6]. At the mucosal surface, polysaccharide (ps)-specific antibodies may inhibit bacterial adherence, promote agglutination and mucociliary clearance, and facilitate opsonophagocytosis [7–9], underscoring their potential role in mucosal defense. As such, vaccine-induced mucosal antibodies may contribute to protection against pneumococcal disease, potentially also dependent on the vaccine used.

To prevent pneumococcal disease in older adults, the 23-valent pneumococcal ps vaccine (PPV23) was introduced in the Dutch national immunization program (NIP) in 2020 for adults aged 60–80 years [10]. Intramuscular PPV23 administration is known to elicit systemic antibody responses in older adults and reduce pneumococcal disease risk [11–13]. However, unlike pneumococcal conjugate vaccines (PCVs) typically used in infant immunization schedules, PPV23 has not been shown to reduce nasopharyngeal pneumococcal carriage [14], and data on its impact on mucosal immunity in older adults are scarce [14].

This study evaluates mucosal salivary IgA and IgG responses following intramuscular PPV23 vaccination in Dutch community-dwelling adults aged 72–79 years (the first cohort vaccinated under the NIP in 2020). Moreover, we investigate how mucosal responses relate to systemic antibody responses. These findings will clarify the potential of PPV23 to induce mucosal immunity and inform future pneumococcal vaccination strategies for aging populations.

## METHODS

### Participants and Sample Collection

This study (NL74843.041.20) was embedded within the Doetinchem Cohort Study (DCS; NL63779.041.17), a prospective observational study in the Netherlands [15, 16]. Of the 188 Dutch community-dwelling adults aged 72–79 years (median age: 75; interquartile range [IQR]: 73.8–77.1; 38% female) enrolled in the PPV23 vaccination cohort within the DCS, paired saliva and serum samples were obtained from 178 individuals, up to 4 weeks prior to, and 4–6 weeks after, primary PPV23 (*Pneumovax 23, MSD*) vaccination, administered intramuscularly as part of the Dutch NIP in 2020. Full inclusion and exclusion criteria are provided in Supplementary Table 1.

Participants were instructed to refrain from eating or drinking 30 minutes before saliva collection. Unstimulated saliva was collected without mechanical or chemical stimulation using 2 Oracol® swabs (*Malvern Medical Developments Ltd*), each held in the mouth for 1 minute [17–19]. Venous blood was collected in serum clotting activator tubes (S-Monovette; *Sarstedt*). Blood and saliva samples were centrifuged, after which the supernatants were collected, aliquoted, and stored at −80°C until further analyses.

The study was approved by the Medical Research Ethics Committee NedMec Utrecht (the Netherlands) and conducted in accordance with the Declaration of Helsinki and the International Council for Harmonisation-Good Clinical Practice (ICH-GCP) guidelines. All participants provided written informed consent. The study is registered at the EU Clinical Trials Register (EudraCT 2020-003620-16) and the Dutch Trial Register (NL74843.041.20).

### Serological Analysis

PPV23 ps-specific IgA and IgG concentrations in preabsorbed saliva and serum were quantified using a fluorescent bead-based multiplex immunoassay (MIA) calibrated against the international human pneumococcal standard 007sp, as described previously [20, 21]. Saliva and serum samples were analyzed at a minimum dilution of 1:4 and 1:10, respectively [17, 20]. Salivary antibody concentrations below the lower limit of detection (LLOD) were assigned a value of half the LLOD for the respective serotype.

To determine whether postvaccination serotype-specific salivary IgA was produced locally at the mucosa, we measured ps-specific IgA antibody-bound to epithelial-derived secretory component (SC) [22] concentrations using a mouse anti-human SC antibody (1:1000 diluted; Sigma-Aldrich) as a primary antibody and a R-phycoerythrin-conjugated goat anti-mouse IgG (1:400 diluted; Jackson ImmunoResearch) as a secondary antibody, as described previously [17, 23].

### Statistical Analysis

Salivary ps-specific IgA and IgG geometric mean concentrations (GMCs) with corresponding 95% CIs were calculated for both timepoints. Antibody concentrations were log_10_ transformed prior to statistical analyses. Antibody responses were compared between timepoints using the Wilcoxon signed-rank test. Spearman correlation coefficients were determined to assess the relationship between postvaccination salivary and serum IgA or IgG concentrations and between salivary IgA and SC levels. The effect of age and sex on postvaccination salivary antibody concentrations was analyzed using generalized linear models (GLMs) while correcting for baseline antibody concentrations. Bonferroni corrections were applied for multiple comparisons. A *P*-value of <.05 was considered statistically significant. Analyses were performed using Rstudio (version 4.4.0).

## RESULTS

### Salivary PPV23 ps-Specific IgA and IgG Responses Following PPV23 Vaccination

**Figure 1.**
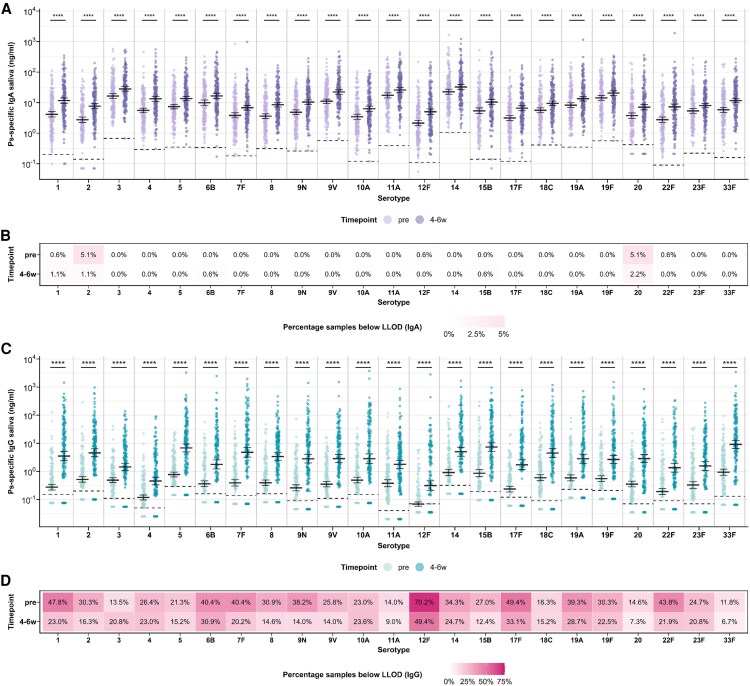
Salivary pneumococcal serotype polysaccharide (ps)-specific IgA and IgG responses following 23-valent pneumococcal polysaccharide vaccine (PPV23) vaccination. Salivary concentrations of ps-specific IgA (*A*) and IgG (*C*) were measured for each of the 23 PPV23 serotypes at baseline (pre) and 4–6 wks postvaccination (4–6 w). Geometric mean concentrations (GMCs) are shown in black, with error bars indicating 95% CIs. Horizontal dashed lines indicate serotype- and immunoglobulin isotype-specific lower limits of detection (LLODs). Antibody concentrations were log_10_ transformed and compared between timepoints using the Wilcoxon signed-rank test; ****P < .0001. Panels *B* (IgA) and *D* (IgG) show the percentage of samples below the serotype-specific LLOD at each time point. Tile color intensity reflects the percentage of samples below the LOD, with darker shading indicating higher percentages.

Prior to vaccination, detectable salivary ps-specific IgA concentrations against PPV23 serotypes were observed for almost all participants (Figure 1*A* and *B*). Baseline IgA GMCs varied between serotypes, ranging from 2.14 ng/mL (95% CI, 1.75–2.61) for serotype 12F to 22.70 ng/mL (95% CI, 18.77–27.44) for serotype 14 (Figure 1*A*; Supplementary Table 2). Four to six weeks after PPV23 vaccination, salivary ps-specific IgA concentrations increased significantly compared to baseline for all 23 serotypes (all *P* < .001). Postvaccination IgA GMCs ranged from 5.03 ng/mL (95% CI, 4.08–6.21) for serotype 12F to 32.61 ng/mL (95% CI, 27.25–39.02) for serotype 14. Fold increases in ps-specific salivary IgA GMCs ranged from 1.44 (95% CI, 1.26–1.64) for serotype 14 to 2.84 (95% CI, 2.38–3.38) for serotype 1 (Supplementary Table 3).

In contrast to IgA, baseline salivary ps-specific IgG concentrations were below the LLOD in a considerable proportion of participants, particularly for serotypes 1 (47.8%), 17F (49.4%), and 12F (70.2%) (Figure 1*C* and *D*). Prevaccination IgG GMCs varied from 0.07 ng/mL (95% CI, .06–.08) for serotype 12F to 0.91 ng/mL (95% CI, .70–1.18) for serotype 14 (Figure 1*C*; Supplementary Table 2). Significant increases in salivary ps-specific IgG concentrations following PPV23 vaccination were observed for all serotypes (all *P* < .001). Fold increases in salivary IgG GMCs ranged from 2.95 (95% CI, 2.20–3.95) for serotype 3 to 12.72 (95% CI, 2.29–17.41) for serotype 1 (Supplementary Table 3). Nonetheless, for certain serotypes, IgG concentrations remained below the LLOD in a substantial number of samples (eg, 23.0%, 33.1%, and 49.4% for serotypes 1, 17F, and 12F, respectively), indicating that a notable proportion of participants did not develop measurable salivary IgG responses (Figure 1*D*).

No significant effects of age or sex were observed on salivary antibody responses following PPV23 vaccination (Supplementary Table 4). Only for serotype 18, increasing age (despite the narrow age range) was associated with reduced salivary IgG responses (*P* = .035). Additionally, serotype 2 and 22F-specific salivary IgG responses were significantly higher in females than males (*P* = .011 and *P* = .015, respectively). Overall, salivary IgA and IgG responses followed similar trends as observed in serum (Supplementary Figure 1 and Table 2).

### Correlations Between Salivary and Serum Antibody Responses and Secretory Component Levels Following PPV23 Vaccination

**Figure 2.**
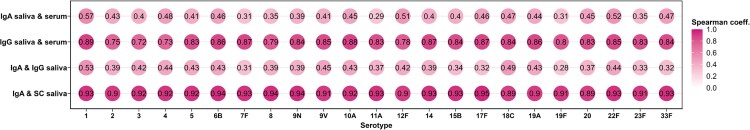
Correlations between postvaccination salivary and serum pneumococcal polysaccharide–specific IgA and IgG responses and salivary secretory component (SC) levels following 23-valent pneumococcal polysaccharide vaccine (PPV23) vaccination. Spearman correlation coefficients and corresponding significance levels are shown for pairwise comparisons between PPV23 ps-specific antibody concentrations measured 4–6 wks postvaccination. Correlations were assessed between salivary and serum IgA levels, salivary and serum IgG levels, salivary IgA and IgG levels, and salivary IgA and SC mean fluorescence intensities (MFIs). Color intensity reflects the strength of the Spearman correlation coefficient (rho). All correlations were statistically significant (*P* < .001 after Bonferroni correction).

Significant but generally weak-to-moderate correlations were observed between postvaccination salivary and serum ps-specific IgA concentrations (rho = 0.29–0.57; all *P* < .001), as well as between salivary ps-specific IgA and IgG concentrations (rho = 0.31–0.53; all *P* < .001; Figure 2). In contrast, strong correlations were found between postvaccination salivary and serum ps-specific IgG concentrations (rho = 0.72–0.89; all *P* < .001) and between salivary ps-specific IgA concentrations and mean fluorescence intensity (MFI) levels of the secretory component (rho = 0.87–0.95; all *P* < .001).

## DISCUSSION

In this study, we demonstrated that intramuscular PPV23 vaccination induces salivary ps-specific IgA and IgG responses in older adults aged 72–79 years. Salivary IgA responses were observed in virtually all participants, whereas serotype-specific salivary IgG responses remained undetectable in ∼7%–49% of participants.

Notably, the presence of ps-specific salivary antibodies prior to vaccination reflects preexisting mucosal immunity likely due to lifelong natural exposure to circulating pneumococcal serotypes as reported by others [24, 25] While the significance of ps-specific antibodies for protection against disease remains unclear, previous research suggests that concentrations are generally lower in older adults than in younger adults and that a decline in local immunity may contribute to increased infection risk in this age group [26].

Data on ps-specific mucosal antibody responses following PPV23 vaccination are scarce. In contrast to our findings, Heaney et al [26] reported that PPV23-vaccinated older adults did not demonstrate higher salivary ps-specific IgA or IgG concentrations as compared to vaccine-naive counterparts, whereas higher antibody levels were observed in PPV23-vaccinated younger adults compared to their unvaccinated peers [26]. However, these findings should be interpreted with caution, as antibody responses were evaluated at variable time points, up to 6 years postvaccination in older adults and 4 years in younger adults, and the sample sizes were small.

Post-PPV23 salivary IgA concentrations correlated strongly with SC levels, a marker of local production by mucosal plasma cells. Dimeric IgA is actively transported across epithelial cells via the polymeric immunoglobulin receptor (pIgR), resulting in SC-bound IgA (also referred to as secretory IgA [sIgA]) [27]. Consistent with this, the correlation between salivary and serum IgA concentrations was weak, further supporting a predominantly mucosal origin of salivary IgA. In contrast, the strong correlation between salivary and serum IgG concentrations suggests that salivary IgG primarily originates from serum (likely via FcRn-mediated translocation) [28], rather than local production.

The salivary antibody response to PPV23 is an indicator of mucosal immunity, which may play a role in preventing pneumococcal colonization and subsequent transmission or disease. Previous studies have demonstrated a functional role for mucosal antibodies (specifically IgG) in limiting colonization, thereby potentially reducing the risk of transmission and disease [6,29] , supporting the relevance of our findings. While it remains to be confirmed whether PPV23-induced salivary antibodies observed here confer protection, these results provide a strong rationale for further research to explore the functional contribution of mucosal antibodies in limiting pneumococcal acquisition, colonization, and disease in older adults.

This study has limitations: The short follow-up period (4–6 weeks) precluded assessment of the persistence of PPV23-induced mucosal antibody responses, and the absence of a younger comparator group limits conclusions regarding age-specific effects on the induction of mucosal immunity. Moreover, directly comparing mucosal antibody responses after PPV23 and PCV vaccination could help clarify why only PCVs have been shown to reduce pneumococcal carriage.

In conclusion, intramuscular PPV23 vaccination elicits salivary ps-specific IgA and IgG responses in the majority of older adults aged 72–79 years. While salivary IgA responses appear to be produced mucosally, salivary IgG probably is largely serum derived. Future research evaluating mucosal antibody responses beyond the first month following pneumococcal vaccination, as well as direct comparisons with younger age groups, is warranted to better contextualize our results.

## Supplementary Material

ofag273_Supplementary_Data

## Supplementary Data

Supplementary materials are available at *Open Forum Infectious Diseases* online. Consisting of data provided by the authors to benefit the reader, the posted materials are not copyedited and are the sole responsibility of the authors, so questions or comments should be addressed to the corresponding author.
