# Supplementary material for: Induction of Salivary IgA and IgG Responses by Parenteral PPV23 Vaccination in Older Adults
Source: Open Forum Infect Dis. 2026 May 4;13(5):ofag273. doi: 10.1093/ofid/ofag273 (PMC13195296; doi:10.1093/ofid/ofag273)

SUPPLEMENTARY INFORMATION

Induction of salivary IgA and IgG responses by parenteral PPV23 vaccination in older adults

Maxime Visser[1,2], Irina Tcherniaeva[1], Lia de Rond[1], Mary-lène de Zeeuw-Brouwer[1], Monique W.M. Verschuren[3,4], Susan H.S.J. Picavet[3], Marien .I. de Jonge[2], Gerco den Hartog[1,2], Anne-Marie Buisman[1]

[1] Centre for Immunology of Infectious Diseases and Vaccines, National Institute for Public Health and the Environment, RIVM, Bilthoven, The Netherlands.

[2] Laboratory of Medical Immunology, Radboud university medical center, Nijmegen, The Netherlands.

[3] Centre for Nutrition, Prevention and Health Services, National Institute for Public Health and the Environment, RIVM, Bilthoven, The Netherlands

[4] Julius Center for Health Sciences and Primary Care, University Medical Center Utrecht, Utrecht, The Netherlands

Corresponding author: Anne-Marie Buisman ; annemarie.buisman@rivm.nl; National Institute for Public Health and the Environment (RIVM), Antonie van Leeuwenhoeklaan 9, 3721 MA, Bilthoven, The Netherlands.

Supplementary table 1. In- and exclusion criteria.

Inclusion criteria

- Participated in round 6 (2013-2017) of the Doetichem Cohort Study;
- Have a general practitioner (GP) in the municipality of Doetichem or Gaanderen;
- Must be willing to receive the PPV23 vaccine facilitated via the Dutch National immunization program;

Exclusion criteria

- Previous pneumococcal vaccination;
- Known or suspected allergy to any of the vaccine components or having experienced a previous severe adverse reaction to any vaccine;
- Receipt of any high-dose (≥ 20 mg of prednisone daily or equivalent) daily corticosteroids (locally applied including inhaled steroids are acceptable) within 2 weeks of study entry, or, as medically prescribed, within two weeks after the vaccination;
- Repeated use of any high dose of corticosteroids (a dose of > 30 mg of prednisone or equivalent per day for multiple days) in the last month;
- Receipt of an organ- or bone marrow transplant during the last 5 years;
- Having an anatomical or functional asplenia;
- Receipt of blood products or immunoglobulin, within one month of study entry;
- Known or suspected coagulation disorder that in the judgement of the investigator would contraindicate against receiving an intramuscular injection or undergo frequent blood sampling;
- Known to be positive for human immunodeficiency virus (HIV), and/or hepatitis C virus (HCV) and/or hepatitis B virus (HBV) (presence of HBsAg or anti HBc antibodies).
- Start of a treatment with chemotherapy during the last three months;

Temporary exclusion criteria

- If a subject has an elevated body temperature fewer than 48 hours before a visit with blood sampling the visit should be postponed until this criterion is no longer met;
- Receipt of any other vaccine(s) 2 weeks before a blood collection.

Withdrawal criteria during study

- Receipt of any high-dose (≥ 20 mg of prednisone daily or equivalent) daily corticosteroids (local incl. inhaled steroids are acceptable) within 2 weeks of study entry, or, as medically prescribed, within two weeks after the vaccination;
- Receipt of blood products or immunoglobulins.

Supplementary table 2. PPV23 serotype-specific IgA and IgG geometric mean concentrations (GMCs) in saliva and serum before and four-to-six weeks after PPV23 vaccination

	IgA saliva		IgA serum		IgG saliva		IgG serum	
**Sero-type**	**Timepoint Baseline**	**4-6 weeks**	**baseline**	**4-6 weeks**	**baseline**	**4-6 weeks**	**baseline**	**4-6 weeks**
**1**	4.13 [3.38-5.04]	11.71 [9.56-14.35]****	24.27 [20.35-28.95]	484.72 [386.09-608.55]****	0.28 [0.22-0.35]	3.50 [2.37-5.18]****	204.93 [160.20-262.16]	3624.82 [2652.08-4954.34]****
**2**	2.75 [2.23-3.39]	7.64 [6.32-9.24]****	10.48 [9.36-11.74]	205.67 [163.60-258.56]****	0.52 [0.41-0.64]	4.56 [3.29-6.34]****	472.71 [369.27-605.13]	6764.40 [5345.94-8559.24]****
**3**	16.47 [13.81-19.64]	28.11 [23.69-33.35]****	120.24 [102.68-140.81]	318.71 [263.14-386.01]****	0.49 [0.40-0.59]	1.44 [1.06-1.95]****	214.35 [173.54-264.76]	1387.95 [1097.98-1754.48]****
**4**	5.57 [4.68-6.62]	13.40 [11.09-16.19]****	23.47 [20.25-27.21]	163.59 [127.65-209.64]****	0.12 [0.10-0.14]	0.46 [0.33-0.62]****	54.06 [43.87-66.62]	440.59 [335.67-578.32]****
**5**	7.37 [6.24-8.71]	13.61 [11.49-16.10]****	27.25 [23.75-31.26]	250.23 [198.03-316.19]****	0.77 [0.63-0.94]	6.82 [4.91-9.48]****	621.35 [508.18-759.72]	7505.66 [5733.86-9824.95]****
**6B**	9.93 [8.11-12.15]	16.59 [13.57-20.27]****	31.41 [26.23-37.62]	193.13 [154.76-241.02]****	0.36 [0.29-0.46]	1.77 [1.22-2.58]****	185.83 [140.64-245.54]	1779.75 [1258.04-2517.81]****
**7F**	3.83 [3.16-4.63]	6.78 [5.70-8.06]****	18.31 [15.85-21.15]	156.81 [126.63-194.19]****	0.39 [0.30-0.51]	4.75 [3.21-7.04]****	411.99 [318.31-533.26]	6353.04 [4698.05-8591.05]****
**8**	3.61 [3.02-4.32]	8.53 [7.19-10.12]****	16.22 [13.88-18.94]	152.51 [124.29-187.15]****	0.39 [0.32-0.49]	3.38 [2.49-4.59]****	216.93 [171.95-273.67]	3087.67 [2466.87-3864.68]****
**9N**	4.85 [4.06-5.80]	10.38 [8.74-12.33]****	13.77 [11.79-16.09]	225.22 [168.90-300.31]****	0.26 [0.20-0.33]	2.81 [1.97-4.00]****	184.61 [140.93-241.84]	3147.53 [2333.05-4246.36]****
**9V**	11.08 [9.35-13.13]	22.31 [18.98-26.24]****	35.67 [30.69-41.45]	289.73 [239.50-350.49]****	0.35 [0.28-0.44]	2.84 [2.03-3.96]****	235.87 [190.45-292.13]	2783.60 [2121.23-3652.81]****
**10A**	3.45 [2.85-4.18]	6.30 [5.19-7.65]****	18.71 [15.59-22.46]	220.72 [178.37-273.13]****	0.49 [0.39-0.62]	2.83 [1.89-4.22]****	297.27 [225.24-392.34]	3763.96 [2673.69-5298.82]****
**11A**	17.34 [14.31-21.01]	25.89 [21.86-30.67]****	60.97 [50.71-73.31]	235.40 [192.18-288.33]****	0.38 [0.28-0.50]	1.78 [1.29-2.45]****	312.20 [242.83-401.40]	1860.56 [1429.49-2421.62]****
**12F**	2.14 [1.75-2.61]	5.03 [4.08-6.21]****	5.86 [4.84-7.09]	180.42 [144.98-224.51]****	0.07 [0.06-0.08]	0.31 [0.21-0.46]****	33.92 [27.10-42.45]	375.58 [266.00-530.30]****
**14**	22.70 [18.77-27.44]	32.61 [27.25-39.02]****	57.51 [48.18-68.65]	102.71 [84.04-125.54]****	0.91 [0.70-1.18]	5.02 [3.48-7.24]****	506.33 [379.20-676.08]	3911.19 [2732.17-5599.00]****
**15B**	5.39 [4.36-6.67]	10.43 [8.54-12.74]****	17.21 [14.42-20.55]	773.73 [644.43-928.99]****	0.86 [0.64-1.15]	7.35 [5.18-10.43]****	577.93 [426.55-783.02]	6925.82 [5077.45-9447.06]****
**17F**	3.13 [2.59-3.79]	6.57 [5.46-7.91]****	11.52 [9.83-13.50]	246.08 [200.14-302.55]****	0.24 [0.19-0.30]	1.72 [1.15-2.56]****	182.21 [136.43-243.36]	2748.55 [1998.65-3779.82]****
**18C**	5.60 [4.67-6.73]	9.45 [7.97-11.21]****	22.48 [19.38-26.09]	270.98 [222.11-330.60]****	0.59 [0.46-0.76]	4.48 [3.09-6.51]****	565.84 [437.15-732.40]	5837.36 [4350.30-7832.74]****
**19A**	8.25 [6.86-9.93]	13.57 [11.34-16.23]****	43.77 [35.36-54.17]	316.73 [259.93-385.94]****	0.59 [0.46-0.75]	2.81 [1.93-4.11]****	714.36 [555.63-918.42]	4711.62 [3492.83-6355.70]****
**19F**	14.28 [11.97-17.05]	20.56 [17.42-24.26]****	55.38 [47.12-65.09]	211.62 [165.62-270.41]****	0.55 [0.44-0.69]	2.65 [1.89-3.74]****	514.50 [409.47-646.47]	3691.41 [2784.12-4894.37]****
**20**	3.76 [3.07-4.61]	7.07 [5.79-8.63]****	18.62 [15.69-22.10]	198.40 [164.12-239.84]****	0.35 [0.28-0.44]	2.86 [2.06-3.95]****	610.06 [497.06-748.74]	4353.68 [3425.96-5532.60]****
**22F**	2.77 [2.26-3.40]	7.24 [5.83-8.99]****	12.14 [9.79-15.06]	225.67 [187.59-271.48]****	0.19 [0.15-0.25]	1.36 [0.94-1.95]****	98.61 [73.98-131.44]	1335.06 [995.12-1791.13]****
**23F**	5.39 [4.48-6.47]	7.98 [6.70-9.50]****	24.37 [20.79-28.57]	208.83 [170.50-255.77]****	0.33 [0.25-0.43]	1.57 [1.09-2.25]****	243.68 [189.27-313.75]	1711.59 [1275.24-2297.25]****
**33F**	5.83 [4.81-7.05]	11.71 [9.80-13.98]****	19.70 [16.57-23.43]	364.95 [297.32-447.96]****	0.94 [0.73-1.20]	9.13 [6.58-12.68]****	797.65 [623.37-1020.65]	8658.84 [6577.45-11398.87]****

PPV23 ps-specific IgA and IgG geometric mean concentrations (GMCs [95%CI]; ng/ml) were determined in saliva and serum at baseline and at four-to-six weeks after PPV23 vaccination. Antibody GMCs were compared between timepoints using the Wilcoxon signed-rank test. ****p<0.0001

Supplementary table 3. Fold increase in serotype-specific IgA and IgG geometric mean concentrations in saliva and serum from pre-vaccination to 4-6 weeks post-vaccination.

	**Fold increase in GMC [95%CI]**			
**Serotype**	**IgA saliva**	**IgA serum**	**IgG saliva**	**IgG serum**
**1**	2.84 [2.38-3.38]	19.97 [16.54-24.11]	12.72 [9.29-17.41]	17.69 [14.31-21.87]
**2**	2.78 [2.34-3.31]	21.05 [17.12-25.90]	8.84 [6.69-11.69]	14.31 [11.88-17.24]
**3**	1.71 [1.47-1.99]	6.43 [5.41-7.65]	2.95 [2.20-3.95]	6.48 [5.26-7.97]
**4**	2.41 [2.05-2.83]	11.55 [9.55-13.95]	3.84 [2.99-4.94]	8.15 [6.79-9.78]
**5**	1.84 [1.60-2.12]	11.63 [9.87-13.69]	8.85 [6.84-11.46]	12.08 [10.11-14.44]
**6B**	1.67 [1.44-1.93]	6.74 [5.56-8.16]	4.91 [3.67-6.56]	9.58 [7.69-11.93]
**7F**	1.77 [1.54-2.04]	10.84 [9.17-12.80]	12.17 [9.04-16.38]	15.42 [12.73-18.68]
**8**	2.36 [2.03-2.75]	13.92 [11.58-16.72]	8.57 [6.51-11.28]	14.23 [11.55-17.54]
**9N**	2.14 [1.84-2.49]	15.16 [12.64-18.19]	10.82 [8.30-14.11]	17.05 [14.03-20.71]
**9V**	2.01 [1.76-2.30]	10.23 [8.67-12.07]	8.12 [6.18-10.68]	11.80 [9.75-14.29]
**10A**	1.83 [1.59-2.09]	10.99 [8.99-13.44]	5.78 [4.18-7.98]	12.66 [10.33-15.52]
**11A**	1.49 [1.31-1.71]	5.23 [4.50-6.07]	4.72 [3.74-5.95]	5.96 [5.06-7.01]
**12F**	2.35 [1.98-2.79]	27.92 [22.45-34.72]	4.55 [3.36-6.16]	11.07 [8.78-13.97]
**14**	1.44 [1.26-1.64]	4.35 [3.65-5.18]	5.51 [4.12-7.35]	7.72 [6.20-9.62]
**15B**	1.93 [1.65-2.26]	11.22 [9.21-13.66]	8.55 [6.43-11.37]	11.98 [9.71-14.79]
**17F**	2.10 [1.78-2.48]	13.62 [11.32-16.38]	7.31 [5.38-9.93]	15.08 [12.40-18.35]
**18C**	1.69 [1.50-1.90]	6.78 [5.80-7.94]	7.56 [5.73-9.97]	10.32 [8.53-12.47]
**19A**	1.64 [1.43-1.89]	5.15 [4.28-6.18]	4.78 [3.67-6.21]	6.60 [5.46-7.96]
**19F**	1.44 [1.27-1.64]	5.23 [4.43-6.17]	4.82 [3.66-6.34]	7.17 [5.93-8.69]
**20**	1.88 [1.64-2.16]	12.64 [10.65-15.00]	8.10 [6.37-10.29]	7.14 [6.09-8.36]
**22F**	2.61 [2.19-3.11]	14.86 [12.08-18.28]	7.05 [5.23-9.51]	13.54 [10.70-17.12]
**23F**	1.48 [1.29-1.70]	4.21 [3.59-4.95]	4.77 [3.65-6.23]	7.02 [5.77-8.55]
**33F**	2.01 [1.73-2.34]	12.49 [10.39-15.01]	9.74 [7.59-12.49]	10.86 [9.01-13.08]

Fold increases in geometric mean concentrations (GMCs) were calculated by dividing 4-6 weeks post-vaccination concentrations divided by baseline concentrations. GMCs are shown with corresponding 95% confidence intervals (95%CIs).

Supplementary table 4. Effect of age and sex on PPV23 ps-specific IgA and IgG responses four to six weeks post-vaccination. Generalized linear models (GLMs) were used to evaluate the effects of sex. and age at vaccination on salivary IgA and IgG responses at four to six weeks (4-6w) following PPV23 vaccination. Models were adjusted for baseline ps-specific IgA/IgG concentrations. Benjamini-Hochberg corrections were applied to account for multiple testing. Significant effects (adjusted (adj.) p<0.05) are indicated in bold.

		IgA		*IgG*	
**Serotype**	**Variable**	**Estimate**	**Adj. p-value**	**Estimate**	**Adj p-value**
**1**	Baseline antibody concentration	**0.623**	**0.000**	**1.008**	**0.000**
	Sex (male)	-0.160	0.058	-0.283	0.100
	Age at vaccination	-0.007	0.687	-0.014	0.702
**2**	Baseline antibody concentration	**0.558**	**0.000**	**0.790**	**0.000**
	Sex (male)	-0.088	0.406	**-0.347**	**0.011**
	Age at vaccination	0.005	0.789	-0.040	0.189
**3**	Baseline antibody concentration	**0.593**	**0.000**	**0.582**	**0.000**
	Sex (female)	-0.089	0.303	-0.304	0.039
	Age at vaccination	0.006	0.762	0.008	0.982
**4**	Baseline antibody concentration	**0.664**	**0.000**	**0.919**	**0.000**
	Sex (male)	-0.047	0.875	-0.103	0.748
	Age at vaccination	0.008	0.875	0.001	0.972
**5**	Baseline antibody concentration	**0.652**	**0.000**	**1.034**	**0.000**
	Sex (male)	-0.071	0.311	-0.152	0.405
	Age at vaccination	-0.012	0.413	-0.014	0.639
**6B**	Baseline antibody concentration	**0.737**	**0.000**	**0.998**	**0.000**
	Sex (male)	-0.012	0.843	-0.072	0.981
	Age at vaccination	-0.005	0.843	-0.001	0.981
**7F**	Baseline antibody concentration	**0.624**	**0.000**	**0.992**	**0.000**
	Sex (male)	-0.100	0.157	-0.082	0.554
	Age at vaccination	0.013	0.473	-0.026	0.554
**8**	Baseline antibody concentration	**0.589**	**0.000**	**0.677**	**0.000**
	Sex (male)	-0.040	0.629	-0.068	0.969
	Age at vaccination	0.016	0.609	0.001	0.969
**9N**	Baseline antibody concentration	**0.615**	**0.000**	**0.934**	**0.000**
	Sex (male)	0.002	0.980	0.023	0.854
	Age at vaccination	-0.010	0.689	-0.012	0.854
**9V**	Baseline antibody concentration	**0.652**	**0.000**	**0.863**	**0.000**
	Sex (male)	0.007	0.893	-0.053	0.675
	Age at vaccination	-0.010	0.595	-0.038	0.288
**10A**	Baseline antibody concentration	**0.750**	**0.000**	**1.003**	**0.000**
	Sex (male)	-0.066	0.357	-0.216	0.299
	Age at vaccination	0.017	0.357	-0.007	0.845
**11A**	Baseline antibody concentration	**0.645**	**0.000**	**0.811**	**0.000**
	Sex (male)	0.000	0.996	-0.033	0.749
	Age at vaccination	0.001	0.996	-0.023	0.504
**12F**	Baseline antibody concentration	**0.692**	**0.000**	**1.277**	**0.000**
	Sex (male)	-0.045	0.691	0.064	0.643
	Age at vaccination	-0.007	0.691	-0.050	0.194
**14**	Baseline antibody concentration	**0.695**	**0.000**	**0.863**	**0.000**
	Sex (male)	-0.039	0.597	-0.147	0.263
	Age at vaccination	-0.007	0.597	-0.066	0.056
**15B**	Baseline antibody concentration	**0.663**	**0.000**	**0.748**	**0.000**
	Sex (male)	-0.063	0.483	-0.083	0.824
	Age at vaccination	0.015	0.483	-0.003	0.928
**17F**	Baseline antibody concentration	**0.587**	**0.000**	**1.075**	**0.000**
	Sex (male)	-0.057	0.638	0.049	0.843
	Age at vaccination	0.012	0.638	-0.007	0.843
**18C**	Baseline antibody concentration	**0.725**	**0.000**	**1.025**	**0.000**
	Sex (male)	-0.006	0.900	-0.096	0.447
	Age at vaccination	-0.010	0.539	**-0.070**	**0.035**
**19A**	Baseline antibody concentration	**0.687**	**0.000**	**1.102**	**0.000**
	Sex (male)	0.003	0.959	-0.152	0.414
	Age at vaccination	-0.017	0.335	-0.016	0.582
**19F**	Baseline antibody concentration	**0.670**	**0.000**	**0.909**	**0.000**
	Sex (male)	-0.064	0.330	-0.085	0.613
	Age at vaccination	-0.007	0.571	-0.016	0.613
**20**	Baseline antibody concentration	**0.753**	**0.000**	**0.957**	**0.000**
	Sex (male)	-0.071	0.409	0.018	0.869
	Age at vaccination	-0.009	0.541	-0.037	0.237
**22F**	Baseline antibody concentration	**0.686**	**0.000**	**0.832**	**0.000**
	Sex (male)	-0.135	0.146	**-0.370**	**0.015**
	Age at vaccination	-0.002	0.911	0.006	0.864
**23F**	Baseline antibody concentration	**0.660**	**0.000**	**0.907**	**0.000**
	Sex (male)	-0.047	0.826	-0.147	0.259
	Age at vaccination	-0.001	0.938	-0.034	0.259
**33F**	Baseline antibody concentration	**0.613**	**0.000**	**0.864**	**0.000**
	Sex (male)	-0.047	0.600	-0.043	0.939
	Age at vaccination	-0.007	0.668	0.013	0.939

Supplementary Figure 1. Serum pneumococcal serotype polysaccharide (ps)-specific IgA and IgG responses following PPV23 vaccination. Serum concentrations of ps-specific IgA (**A**) and IgG (**B**) were measured for each of the 23 PPV23 serotypes at baseline (pre) and 4–6 weeks post-vaccination (4–6w). Geometric mean concentrations (GMCs) are shown in black, with error bars indicating 95% confidence intervals. Horizontal dashed lines indicate serotype- and immunoglobulin isotype-specific lower limits of detection (LLOD). Antibody concentrations were $\log_{10}$-transformed and compared between timepoints using the Wilcoxon signed-rank test; ****p<0.0001.

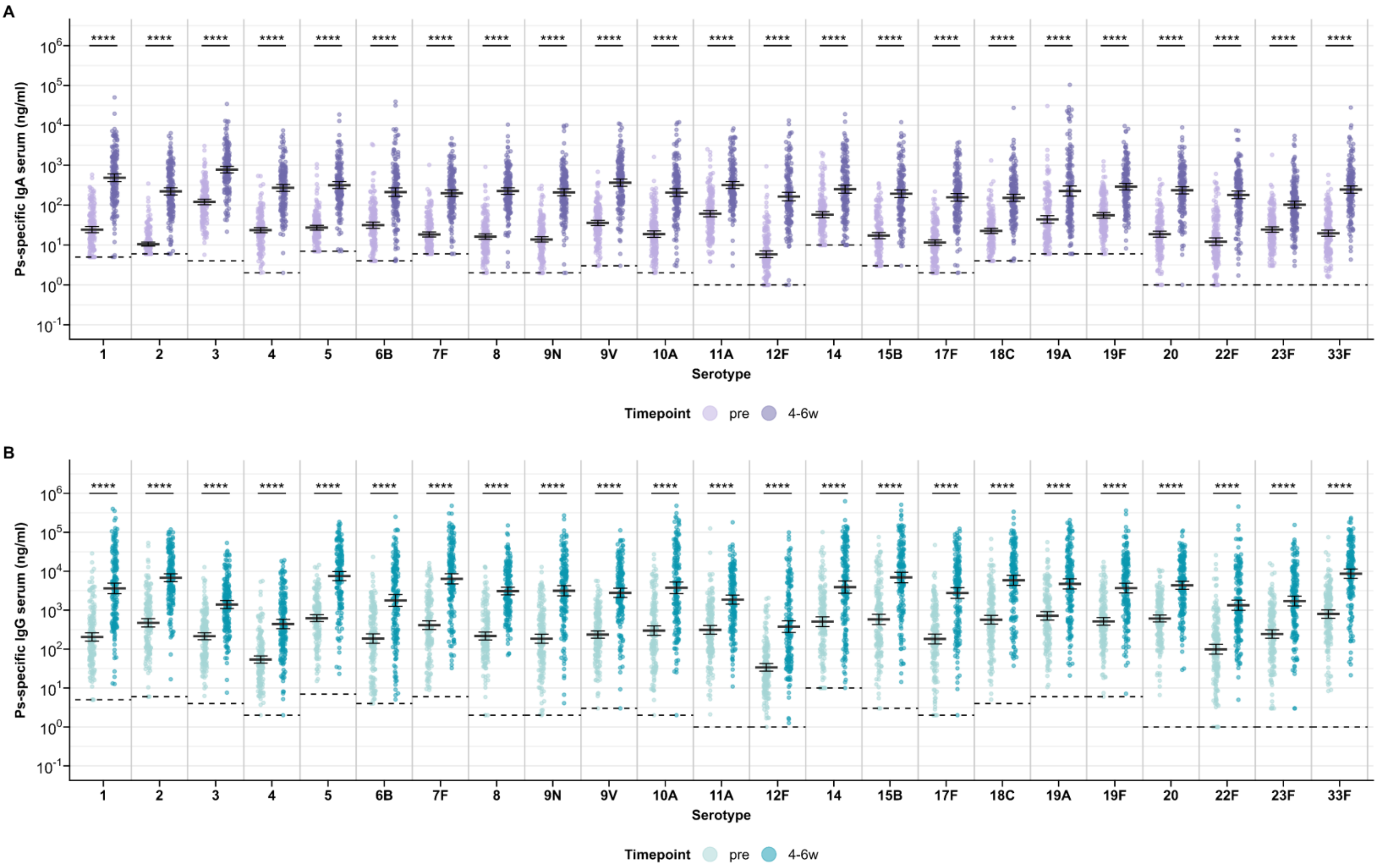

Supplement: ofag273_Supplementary_Data [file ofag273_supplementary_data.pdf]
